# *Acinetobacter baumannii*, *Klebsiella pneumoniae* and *Elizabethkingia miricola* isolated from wastewater have biodegradable activity against fluoroquinolone

**DOI:** 10.1007/s11274-022-03367-5

**Published:** 2022-08-16

**Authors:** Reham Alaa Eldin Shaker, Yosra Ibrahim Nagy, Mina E. Adly, Rania Abdelmonem Khattab, Yasser M. Ragab

**Affiliations:** 1 Department of Microbiology and Immunology, Faculty of Pharmacy, Cairo University, Kasr Al-Aini, Cairo, 11562 Egypt; 2 Department of Pharmaceutical Organic Chemistry, Faculty of Pharmacy, Cairo University, Kasr Al-Aini, Cairo, 11562 Egypt

**Keywords:** Biodegradation, Ciprofloxacin, Fluoroquinolones pollution, Fluoroquinolones, Levofloxacin, LC-MS-MS metabolites

## Abstract

Ciprofloxacin (CIP) and levofloxacin (LEV), widely used fluoroquinolone antibiotics, are often found in sewage from the sewage treatment plants and marine environment. In this study, CIP and LEV biodegrading bacterial consortia were obtained from industrial wastewater. Microorganisms in these consortia were identified as *Acinetobacter baumannii* (*A. baumannii*), *Klebsiella pneumoniae* (*K. pneumoniae*) and *Elizabethkingia miricola* (*E. miricola*)*.* The impacts of the critical operating parameters on the elimination of CIP and LEV by bacterial consortia have been investigated and optimized to achieve the maximum levels of CIP and LEV biodegradation. Using liquid chromatography with tandem mass spectrometry (LC-MS-MS), possible degradation pathways for CIP and LEV were suggested by analyzing the intermediate degradation products. The role of the enzymes fluoroquinolone-acetylating aminoglycoside (6′-N-acetyltransferase) and cytochrome P450 (CYP450) in the breakdown of fluoroquinolones (FQs) was investigated as well. According to our findings, various biodegradation mechanisms have been suggested, including cleavage of piperazine ring, substitution of F atom, hydroxylation, decarboxylation, and acetylation, as the main biotransformation reactions. This study discovers the ability of non-reported bacterial strains to biodegrade both CIP and LEV as a sole carbon source, providing new insights into the biodegradation of CIP and LEV.

**Graphical abstract:**

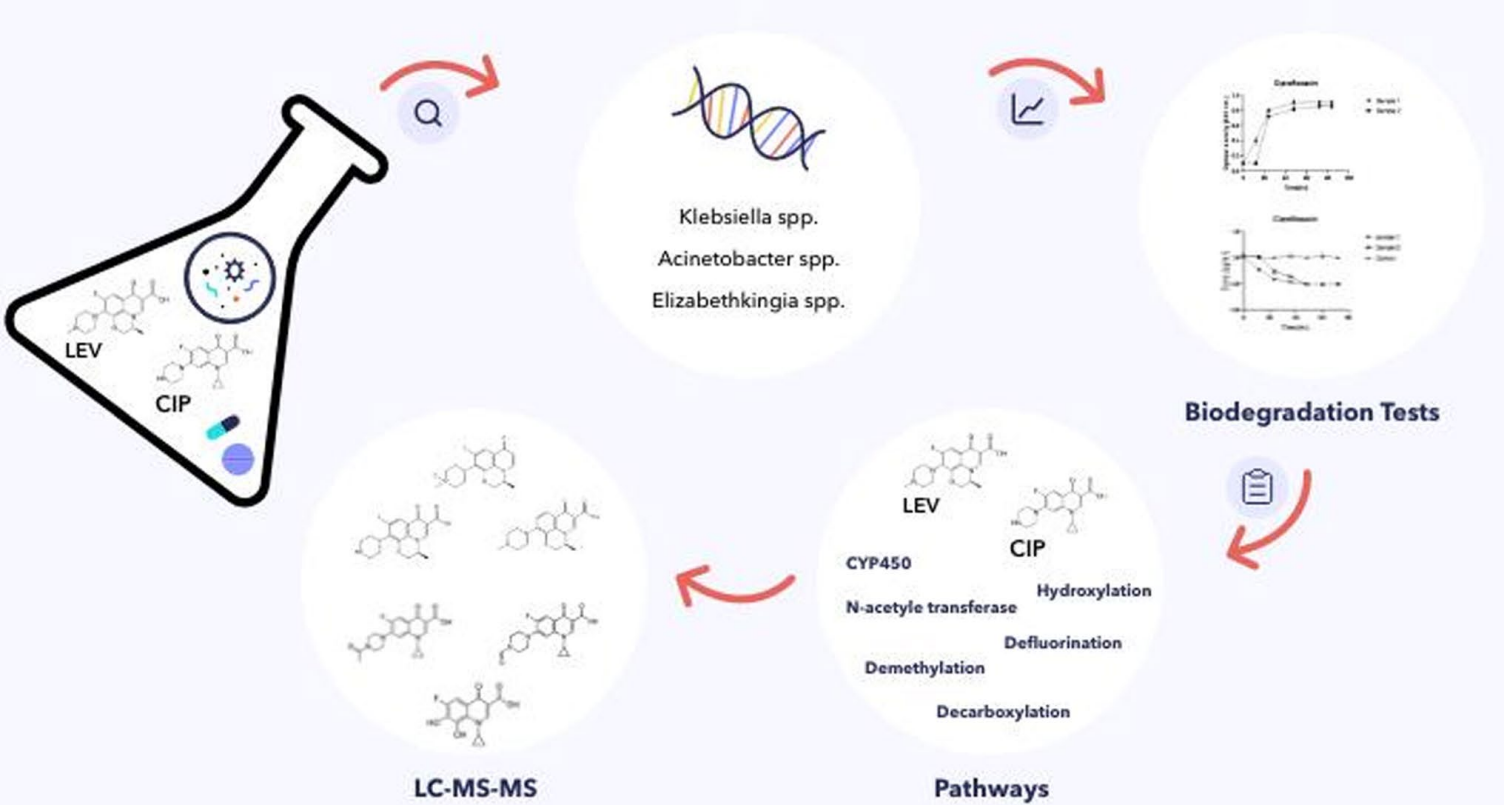

**Supplementary Information:**

The online version contains supplementary material available at 10.1007/s11274-022-03367-5.

## Introduction

FQs are synthetic antibiotics that have been extensively utilized in medical and veterinary medicine. The FQs are constantly used off label as a growth enhancer in animals culture (Kumar et al. 2005). Inhibition of enzymes essential to DNA replication is their principle mode of action (Blondeau 2004). FQs are incompletely metabolized by humans and animals. Besides, up to 90% of these drugs are excreted unchanged in urine and feces (Jelic et al. 2011). Residual FQs can pass easily to the terrestrial environment through the application of antimicrobial laced manure and are easily transferred to the marine environment through wastewater disposal (Rusu et al. 2015). The antimicrobial resistant bacteria might emerge from prolonged environmental exposure to small concentration of antimicrobials (Oliveira et al. 2018). Thus, widespread and inappropriate use of antimicrobials in veterinary and human practice has resulted in the development of bacteria resistant to antimicrobials among pathogenic bacteria, making present antimicrobials ineffectual against common infectious diseases (Homem and Santos 2011).

A number of methods including physical–chemical such as advanced oxidation processes (De Witte et al. 2009; Giri and Golder 2014), sorption by special materials (Peng et al. 2015; Zhao et al. 2016), and photo degradation (Babic et al. 2013) have been adopted to remove CIP. However, these methods possess many drawbacks such as high sludge production, management of the oxidants release of volatile compounds, technical constraints and formation of by-products (Crini and Lichtfouse 2019). An alternative to physical and chemical treatments is the use of living micro-organisms to clean up these antimicrobials. Bioremediation uses microbes to remove complex hazardous substances or break them down into non-toxic or less toxic substances. The process is cost economic than other methods (Azubuike et al. 2016). It’s the most efficient, economical, environmental friendly approach to manage the contaminated environment (Fischer and Majewsky 2014).

The antibiotic CIP is ubiquitous in the environment as a result of its high stability and resistance to degradation. CIP biodegradation has been reported in the literature, however, only a few microbial species can degrade CIP (Pan et al. 2018). Various bacteria were investigated for their CIP biodegradation ability, and several degradation products were suggested (Amorim et al. 2014; Jia et al. 2018; Liao et al. 2016; Liyanage and Manage 2018; Pan et al. 2018). As for LEV (Maia et al. 2018; Shu et al. 2021) are the only reported studies on the bacterial biodegradation of LEV to our knowledge. Owing to their toxicity and inhibition of bacterial activity, FQs have become more resistant to biodegradation (Kumar et al. 2005). The accumulation of CIP traces in the environment can lead to the development of antibiotic-resistant bacteria posing a serious health hazard (Felis et al. 2020; Wess et al. 2020; Zhang et al. 2011). Knowing about the microbial community that breaks down CIP and LEV paves the way for better understanding the biological process of FQs dissipation (Jayamani and Cupples 2015).

Therefore, the investigation of the possibility of CIP and LEV biodegradation by microbial species, and the identification of the metabolites/transformation products originated during biodegradation process is the objective of the present study. In addition, the current study sheds some light on the enzymes involved in FQs biodegradation and possible biodegradation pathways.

## Materials and methods

CIP hydrochloride (HCl) and LEV standards (purity > 99%) were kindly donated by Obour pharmaceutical industries (Cairo, Egypt). Acetonitrile and methanol (HPLC grade) were purchased from Fisher, USA. All other chemicals were of analytical grade. All experiments were conducted in three independent experiments.

### Culture medium

Minimal mineral salt medium (MMSM) was prepared according to method described by Ali and coworkers (Ali et al. 2021). The medium was composed of 10 g FeSO_4_.7H_2_O, 4 g K2HPO4, 4 g KH2PO4, 10 g CaCl_2_.2H_2_O, 100 g MgSO_4_.7H_2_O, 3 g (NH_4_)_2_SO_4_, and trace elements 1 mL^−1^. One liter of distilled water was used as a solvent. Whenever necessary, the MMSM was supplemented with the required concentration of CIP or LEV. When needed, the MMSM was solidified by adding 2% w v^−1^ agar.

### Enrichment, isolation, and maintenance of strains

From various sewage systems of pharmaceutical factories, Cairo, Egypt, wastewater samples were obtained. Two bacterial consortia, each contained two different bacterial strains, were acquired from wastewater containing FQs. Briefly, A volume of 15 mL of wastewater samples were aseptically transferred into 100 mL of MMSM in a conical flask amended with 0.125 mg L^−1^ of either CIP or LEV as a sole organic carbon source, incubated in a rotary shaker incubator at 30 °C and 180 rpm. The microbial culture optical density was regularly monitored at 600 nm (OD600) and if bacterial growth was established (OD600 > 0.7); 10 mL of culture was transported to a new flask containing 100 mL of a fresh MMSM amended with 0.125 mg L^−1^ CIP or LEV. This procedure was repeated 3 consecutive times before 5 mL of the final culture was aseptically inoculated onto MMSM plates supplemented with 0.125 mg L^−1^ of either CIP or LEV. After 48 h incubation at 30 °C, visible colonies were picked and cultured in MMSM supplemented with increasing concentration of CIP or LEV (successively 0.5, 1, 2 mg L^−1^). Samples were selected for further and more detailed study based on the growth rate of microorganisms and the potential to biodegrade FQs as the only source of carbon. Bacterial consortia were preserved in aqueous MMSM (25% glycerol) at − 80 °C (Tamer et al. 2006).

### Identification of FQs biodegrading bacteria in each consortium

The Wizard Genomic DNA purification kit (Promega, USA) was used according to the manufacturer’s manual to extract genomic DNA. Polymerase Chain Reaction (PCR) was used to amplify amplicons of the 16S rRNA gene using primer pair1492r (5′-GGT TAC CTT GTT ACG ACT T-3′) and 27f (5′-AGA GTT TGA TTC TGG CTC AG-3′) (Guo et al. 2005). PCR was performed with conditions 94 °C for 30 s, 52 °C for 30 s, and 72 °C for 90 s for 35 cycles using Veriti thermal cycler (USA) and GoTaq® Flexi DNA polymerase (Promega, USA). PCR products were purified using a QIA quick PCR purification kit (Qiagen, Germany) and sequenced at Macrogen (Seoul, South Korea). The sequences were compared against the available DNA sequences using BLASTN at http://www.ncbi.nlm.nih.gov/BLAST/ maintained by National Center of Biotechnology Information (NCBI).

### Determination of the minimum inhibitory concentration (MIC) of CIP and LEV for each of the four bacterial isolates

 A sequential two-fold serial micro dilution broth procedure was adopted according to the method described in (CLSI 2021) with CIP and LEV concentration ranging from 1056 to 0.5 mg L^−1^. The plates were incubated at 37 °C for 24 h. The lowest concentration of the antimicrobial agent that resulted in complete inhibition of visible growth was considered the MIC.

### FQs biodegradation assays

For each bacterial consortium, the inoculum was prepared by re-suspending the aqueous MMSM glycerol stock in 50 mL of MMSM media supplemented with CIP or LEV as a sole carbon source followed by 18 h incubation at 37 °C and rpm 180. A volume of 5 mL of the overnight bacterial suspension was used to inoculate flasks containing 100 mL MMSM (pH 7.0) amended with 1 mg L^−1^ of either CIP or LEV to achieve an OD600 of value 0.125 corresponding to 10^6^–10^7^ CFU mL^−1.^, flasks were then incubated for 3 and up to 28 days at 28 ± 2 °C and at 180 rpm. Samples were periodically withdrawn, and the optical density was measured. If there was a significant increase in the optical density (OD600 > 0.7), it was considered as a positive microbial growth. A volume of 5 mL was then withdrawn, centrifuged at 3000 rpm for 5 min, filtered through 0.22 nm nylon syringe filter and stored at − 20 °C for further HPLC and phytotoxicity analyses. Negative controls were conducted by using MMSM with the FQs without microbial inoculation and positive controls were done by using MMSM inoculated with the bacterial consortium without FQs addition. The ability of the individual bacterial isolates for biodegradation of either CIP or LEV was also assessed using the previously mentioned conditions. The maximum concentration of FQs degraded by the two bacterial consortia was determined using MMSM supplemented with different concentrations of CIP or LEV ranging from (1–10 mg L ^−1^) as a sole carbon source.

### Optimization of degradation process

A factorial design was used to determine the optimum conditions for the biodegradation process of FQs. Three factors were tested: pH (5 and7), UV application (UV B), Rpm (180 and 210) and time (zero, 24 and 48 h). Minitab 18 program (USA) (www.minitab.com) was used for designing the factorial design and a total of 16 runs were analyzed.

## Analytical methods

### Chromatographic analysis of LEV and CIP HCl biodegradation

A rapid high-performance liquid chromatography method (Waters alliance 2690 series), using Diode Array Detector DAD-3000 (RS), was adopted for measuring the concentrations of CIP and LEV separately as described in (Czyrski and Szałek 2016) with minor modifications. Briefly, A volume of 100 µl of the previously prepared samples was injected into HPLC column during the run of mobile phase. Chromatographic separation was carried out at room temperature using the column YMC PACK C8 (4.6 X 100 mm, 5 μm, USA). The analysis was employed using isocratic elution and UV detection at 277 and 290 nm for CIP and LEV, respectively. Acetonitrile HPLC grade, potassium di-hydrogen phosphate (6.8 g l^−1^ of water) and 100 μl of triethylamine were injected into the column at a rate of flow 1 mL min^−1^ in a ratio (80:20) as a mobile phase. The mobile phase pH was calibrated at 3 with the use of orthophosphoric acid. Mobile phase was filtered under negative pressure pump through 0.2 µm pore size filter membrane and degassed by ultrasonic generator (Branson 1200, Ultrasonics Cooperation, USA) for 15 min. Samples were recorded on integrator peak areas. The concentration of FQs was calculated by relative peak area. Calibration curves for CIP and LEV were constructed by plotting the relative peak area of FQs versus its concentration.

### Analysis of biodegradation intermediates using mass spectrometry

A method described by Pan and coworkers was adopted (Pan et al. 2018). In brief, samples from the biodegradation assay performed under optimum biodegradation parameters were extracted using equal volumes of ethyl acetate followed by evaporation to dryness. The extracts were dissolved in 3 mL methanol. Ultra-performance liquid chromatography tandem mass spectrometry (UPLC) with a XEVO TQD triple quadruple instrument was utilized to analyze the intermediate metabolites from CIP and LEV biodegradation. The chromatographic separation was performed on Waters Corporation, Milford, MA01757 U.S.A, mass spectrometer using ACQUITY UPLC BEH C18 column (1.7 µm − 2.1 × 50 mm) at temperature 30 °C. The mobile phase was composed of solvent A and solvent B where A is acetonitrile containing 0.1% formic acid and B is water containing 0.1% formic acid flowed at rate 0.2 mL min^−1^; 20 µL was injected into the column. The program elution consisted of gradient elution of A from 10 to 100% in 20 min followed by isocratic elution 100% of A for 4 min, followed by 10% of A for 4 min equipped with an electrospray interface (ESI) operated at 350 °C in positive ionization mode, and the ion spray voltage was set as 3 kV. The metabolites were detected and identified by scan analysis from m/z 100 to 1000.

### Determination of bacterial ratio in each consortium during biodegradation process

The assay was done according to (Hazan et al. 2012). In order to find the ratio between each isolate in each consortium during FQs biodegradation, viable count experiments were performed throughout the biodegradation process. A series of tenfold dilutions was prepared in sterile 96 well plate. A volume of 220 µL of each sample, withdrawn at zero, 12, 24, and 48 h, was deposited on the first column of the 96-well micro-plate. Each dilution well received 200 µL of normal saline, 20 µL of each bacterial mixture was successively diluted into 200 µL of normal saline in a serially descending concentration. To each well, 10 μL of diluted bacterial mixture was spotted on brain heart agar plates, incubated for 24 h at 30 °C. Dilutions showing 3–30 distinguishable colonies were used for calculating the total microbial count.

### Molecular screening of the aac (6′)-Ib-cr gene variant

The selected isolates were molecularly screened for the gene encoding the fluoroquinolone-acetylating aminoglycoside 6′-N-acetyltransferase (*aac(6')-Ib-cr*). A DNA fragment (482-bp) was amplified using primer 1: (5′-TTGCGA TGCTCTATGAGTGGCTA-3′) and primer 2:(5′-CTCGAATGCCTGGCGTGTTT- 3′) (Park et al. 2006). Primers were chosen to bind to the conserved region among all known *aac (6)-Ib* variants. PCR was performed using Veriti thermal cycler (USA) and GoTaq® Flexi DNA polymerase (Promega, USA). The PCR was performed with conditions 94 °C for 30 s, 54 °C for 30 s, and 72 °C for 90 s for 35 cycles using Veriti thermal cycler (USA) and GoTaq® Flexi DNA polymerase (Promega, USA). The PCR products were gel purified, followed by direct sequencing using primer 3: (5′- CGTCACTC CATACATTGCAA- 3′). Sequences were analyzed by CLC Main Workbench 8 (CLC Bio, Qiagen, Germany) (https://digitalinsights.qiagen.com/) to identify *aac(6)-Ib-cr*, that is deficit from the restriction site of BstF5I found in the wild-type gene.

### Evaluating the role of CYP450 enzymes in FQs degradation

Based on the assay described by Jia et al. (Jia et al. 2018), the CYP450 enzyme effect on the biodegradation of FQs was tested. Briefly, 1-aminobenzotriazole (ABT) was introduced to MMSM at a final concentration of 1 mM. Bacterial solution was utilized to inoculate flasks containing 100 mL MMSM supplemented with CIP or LEV at 1 mg L^−1^ concentration to achieve an OD600 of value 0.125. The flasks were introduced to an orbital shaker (180 rpm). After 48 h of incubation at 30 °C, withdrawn samples (2 mL) were HPLC analyzed as described above.

### Phytotoxicity assay

The assay was carried out in accordance to the protocol represented by (Hassan et al. 2019). Four different concentrations 100%, 50%, 25% and 10% of the previously prepared samples for both CIP and LEV were made. A volume of 3 mL of the prepared dilutions was poured into each Petri dish. Filter papers (5 cm diameter) were carefully placed. Five seeds of *Lepidium sativum* (*L. sativum*) were placed on the soaked filter paper in each dish. Petri dishes were incubated for 3 days at 30 °C in darkness. Phytotoxicity percentage was estimated as the reduction ratio of the average stem length between the test seeds and the control seeds.

## Results

### Isolated bacterial consortia are capable of degrading CIP and LEV

Two bacterial consortia were acquired with the potential to grow on MMSM plates amended with either CIP or LEV at 1 mg L^−1^ individually. Two different colonies arose from each consortium. Analysis of the partial 16S rRNA gene sequence of the two bacterial consortia revealed that one bacterial consortium was composed of *A. baumannii* and *K. pneumoniae* (Genbank accession number: KJ996147.1 and MN860018.1, respectively) and the other consortium was composed of *E. miricola* and *K. pneumoniae* (Genbank accession number: MZ315066.1and MZ389246.1, respectively).

### The individual bacterial isolates are resistant to CIP and LEV

Upon determining the MIC of the individual bacterial isolates of each consortium against both CIP and LEV, it was found that the MIC of CIP for *K. Pneumoniae* and *A. Baumannii* was 16 mg L^−1^ while for *E. miricola* was 8 mg L^−1^. Meanwhile, the MIC of LEV for the four isolates was 8 mg L^−1^.

### The pH affects the biodegradation rate of FQs

**Fig. 1:**
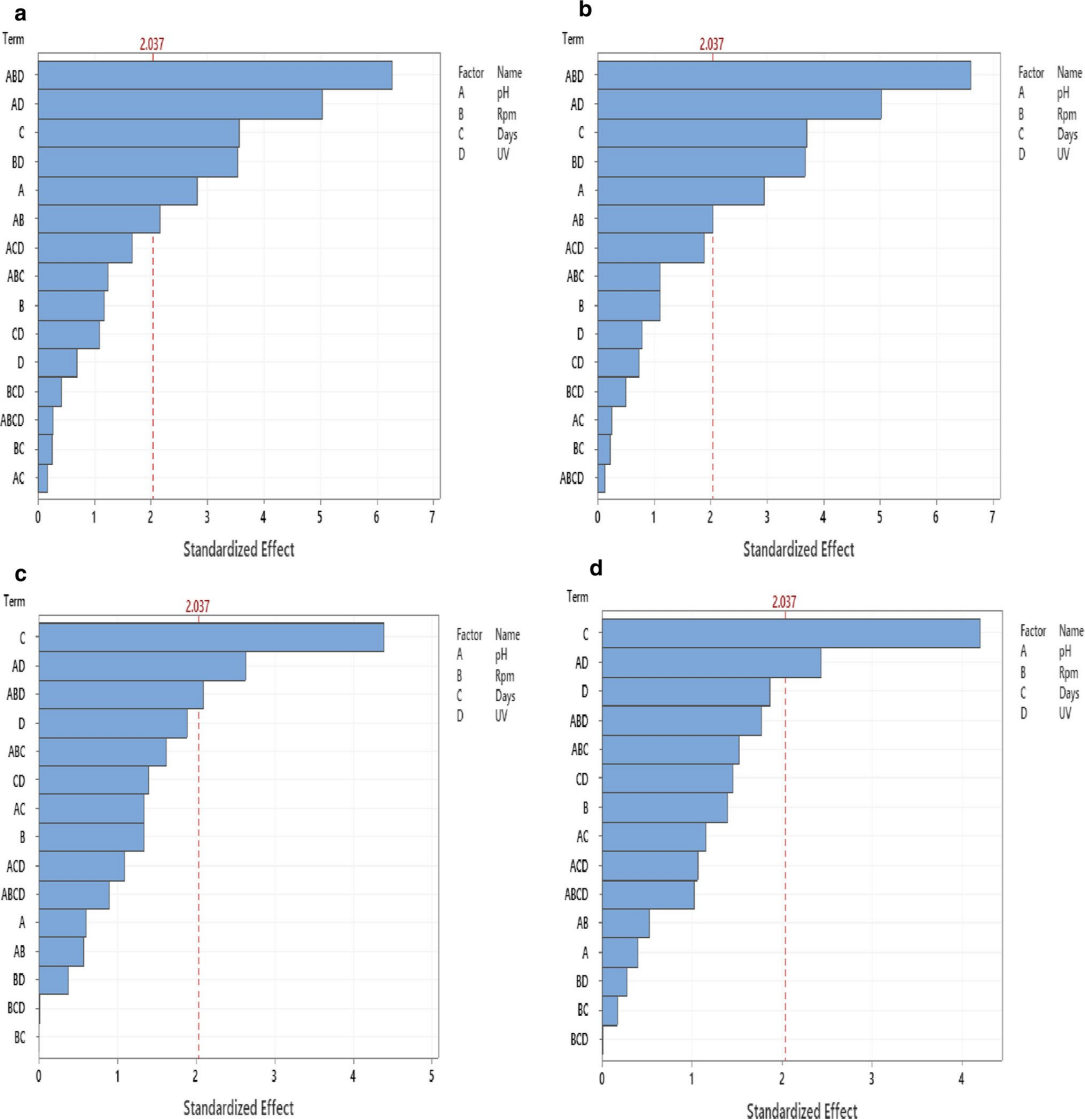
Different variables affect the fluoroquinolones elimination rate. Pareto charts represent the effect of different variables individually and in combinations on the elimination rate of FQS. Pareto charts rank the standardized effects of the tested factors. Bars that cross the reference line are statistically significant. **a** A pareto chart represents the significant variables affecting the biodegradation of CIP by sample 1 (*K. pneumoniae* and *A. baumannii)* (**b)** A pareto chart represents the significant variables affecting the biodegradation of LEV by Sample 1 (*K. pneumoniae* and *A. baumannii*). **c** A pareto chart represents the significant variables affecting the biodegradation of CIP by Sample 2 (*K. pneumoniae* and *E. miricola*). **d** A pareto chart represents the significant variables affecting the biodegradation of LEV by Sample 2 (*K. pneumoniae* and *E. miricola*). Statistical analyses were done at *p*-value < 0.05

By investigating different parameters affecting the FQs biodegradation, the pH factor showed to have a significant effect (*p* < 0.05) on the biodegradation of both CIP and LEV by sample 1 (biodegradation was higher in acidic pH) (Fig. 1a, b), On the contrary, it showed no significant effect on the biodegradation of FQs by sample 2 (Fig. 1c, d). The maximum levels of biodegradation for both CIP and LEV were significantly (*p* < 0.05) attained during day one for both sample 1 and sample 2. No significant difference was displayed by other factors (rpm and UV application). The optimum degradation conditions for both CIP and LEV in sample 1 (*A. baumannii* and *K. pneumoniae*) were temperature 30 °C, pH 5, rpm 180 and without UV application. For sample 2 (*E. miricola* and *K. pneumoniae.*), temperature 30 °C, pH 7, rpm 180 and without UV application represent the optimum conditions for both CIP and LEV biodegradation.

### The bacterial consortia biodegrade FQs individually

**Fig. 2:**
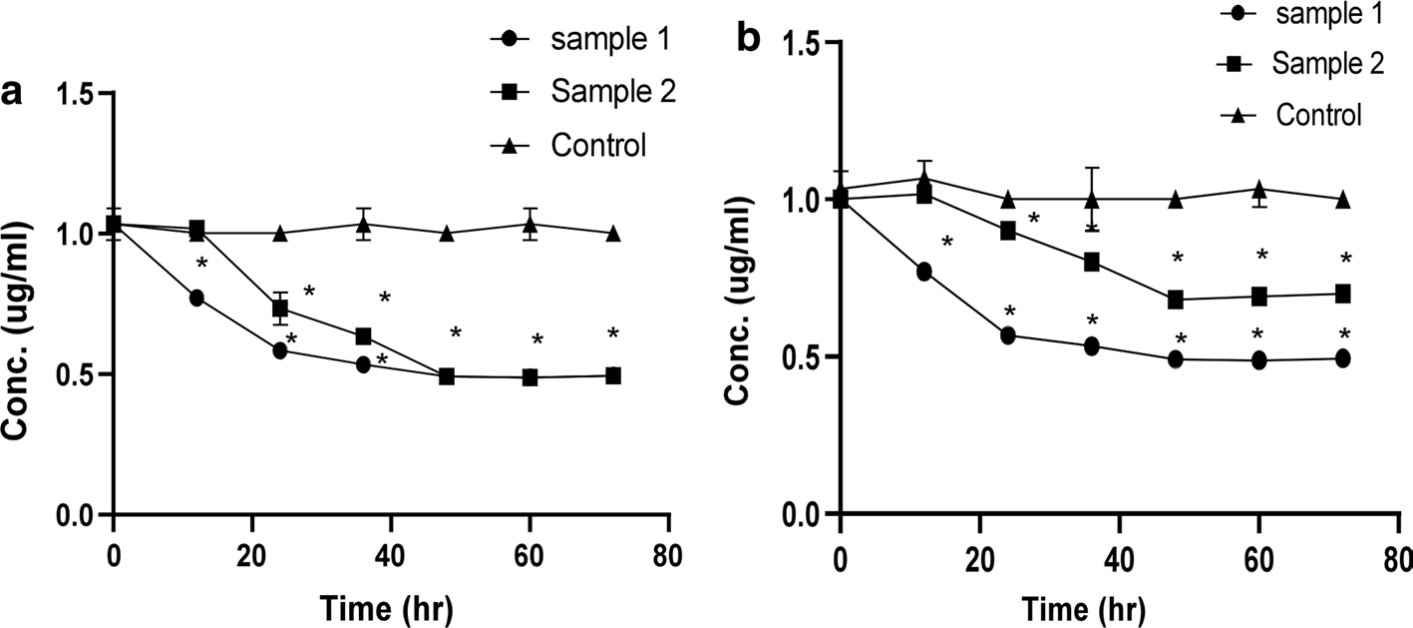
The elimination rate of FQs by bacterial consortia. MMSM was supplemented with CIP or LEV as a sole carbon source. Experiments were conducted under the optimum degradation conditions (30 °C, pH = 5, 180 rpm). **a** A comparison between the biodegradation rate of CIP (1 mg L^−1^) by sample 1 (*K. pneumoniae* and *A. baumannii*) and sample 2 (*K. pneumoniae* and *E. miricola*) and control. **b** A comparison between the biodegradation rate of LEV (1 mg L^−1^) by sample 1 (*K. pneumoniae* and *A. baumannii*) and sample 2 (*K. pneumoniae* and *E. miricola*) and control. Error bars represent SD (n = 3). Statistical analyses were done using two-way ANOVA’s test using graph pad prism version 8 programme (USA). The *indicates significant differences at *p* < 0.05. The figure was generated using CANVA X GIS programme

The quantitative analyses of CIP and LEV biodegradation were monitored by HPLC using DAD detector. CIP and LEV appeared as a single peak at retention time 2.6–2.8 and 2.7–2.8, respectively (Fig. S1). Upon comparing the biodegradation ability of pure bacterial cultures of the individual isolates to that of the consortium, it was found that the biodegradation activity was completely abolished significantly upon using the individual isolates and they should exist as a consortium to exhibit such biodegradation process (Fig.S2). For both bacterial consortia, no significant difference was recorded in the initial concentration of both CIP and LEV 12 h post inoculation. For sample 1, maximum biodegradation levels (50% of the initial concentration) for both CIP and LEV were attained 48 h post inoculation, thereafter, the FQs concentration remained practically unchanged. Meanwhile, 48 h post inoculation, sample two (*E. miricola* and *K. pneumoniae*), was able to biodegrade only 50% and 30% of initial concentration of CIP and LEV, respectively (Fig. 2a, b). The biodegradation metabolites of CIP and LEV are listed in the supporting information Fig. S3 and Fig. S4.

### Different concentrations of FQs affect the rate of biodegradation

**Fig. 3:**
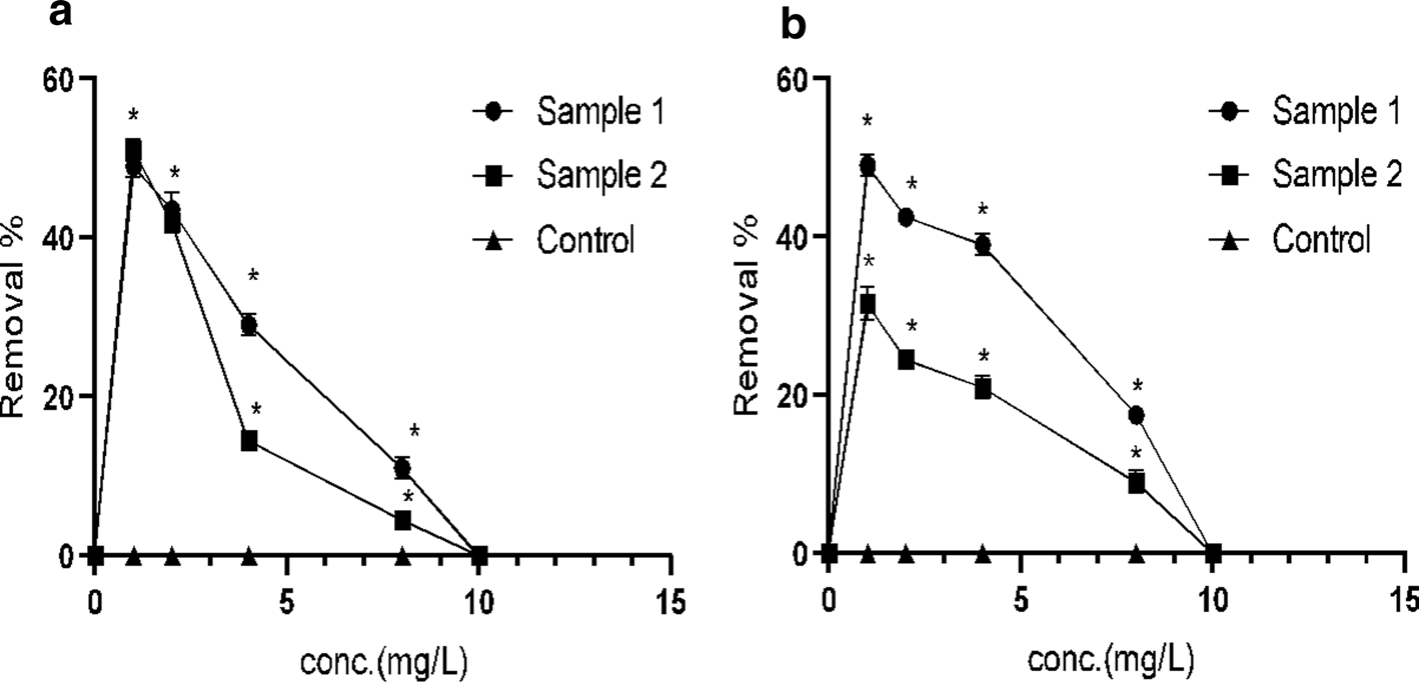
Different concentrations of FQs affect the rate of biodegradation. MMSM was supplemented with CIP or LEV as a sole carbon source. Experiments were conducted under the optimum degradation conditions (30 °C, pH = 5, 180 rpm). **a** A comparison between the biodegradation rate of different conc. of CIP (1–10 mg/L) by sample 1 (*K. pneumoniae* and *A. baumannii*) and sample 2 (*K. pneumoniae* and *E. miricola*) and control. **b** A comparison between the biodegradation rate of different conc. of LEV (1–10 mg/L) by sample 1 (*K. pneumoniae* and *A. baumannii*) and sample 2 (*K. pneumoniae* and *E. miricola*) and control. Values are the means of three independent experiments. Error bars represent standard deviations (n = 3). The analysis was conducted using two way ANOVA’s test using graph pad prism version 8 programme (USA). The *indicates *p* < 0.05

Different concentrations of CIP and LEV were tested to measure the maximum concentration of FQs that can be biodegraded by the bacterial consortium. The two bacterial consortia were able to biodegrade both CIP (Fig. 3a) and LEV (Fig. 3b) up to 8 mg L^−1^ with varying removal rate. As the concentration increased the percentages removal decreased.

### The relative abundance of each isolate of the two bacterial consortia varies during FQS biodegradation

**Fig. 4:**
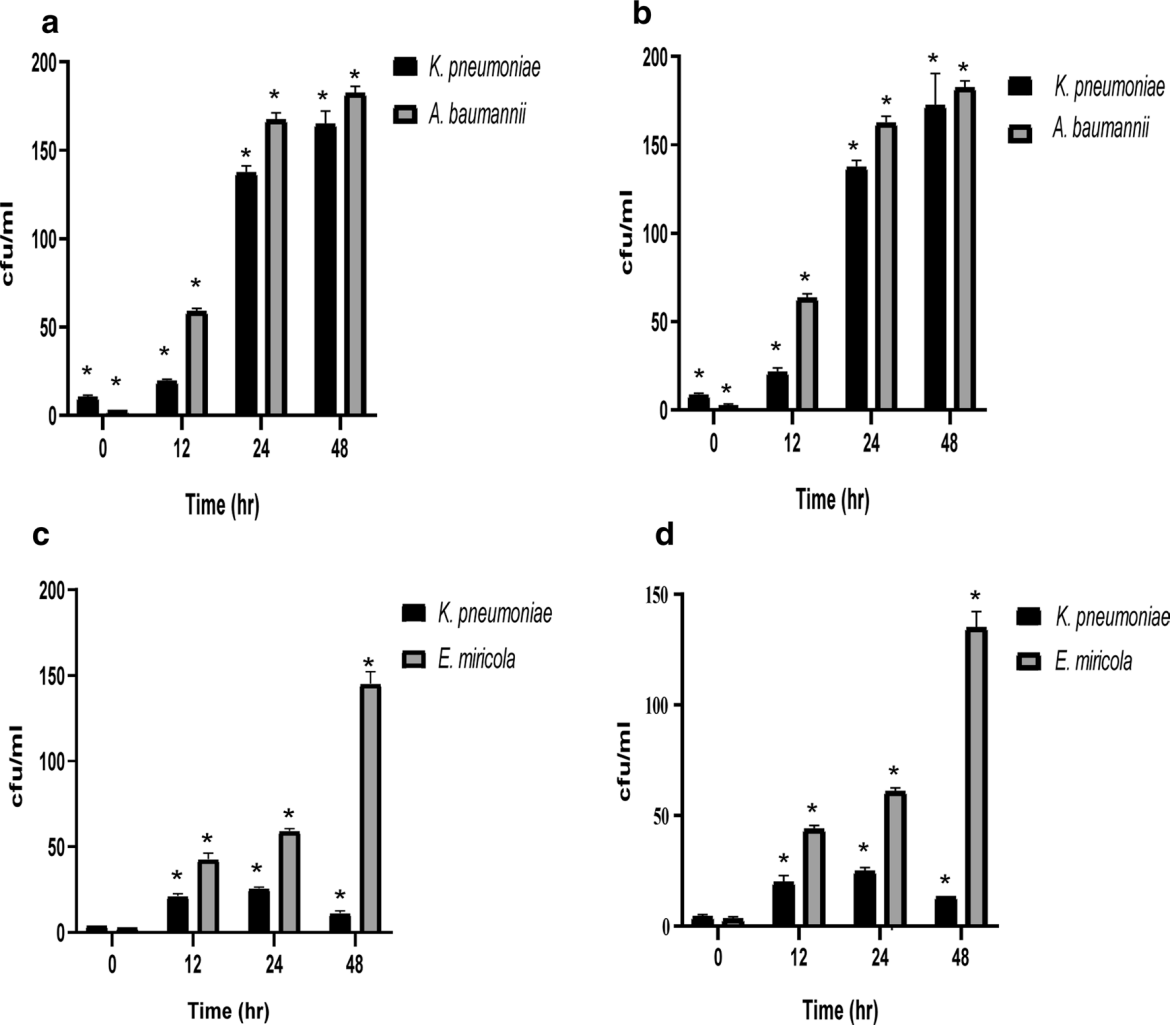
The relative abundance of each isolate of the two bacterial consortia varies during FQs biodegradation. Viable count experiment was performed during the biodegradation process. (**a**) and (**b)** A comparison between the recorded CFU of sample 1 isolates *K. pneumoniae* and *A. baumannii* during the biodegradation of CIP and LEV, respectively. (**c**) and (**d**) A comparison between the recorded CFU of sample 2 isolates *K. pneumoniae* and *E. miricola* during the biodegradation of CIP and LEV, respectively. The analysis was conducted using two-way ANOVA’s test using graph pad prism version 8 programme (USA). The *indicates significant differences, *p* < 0.05. The figure was generated using CANVA X GIS programme

Each dilution containing (3–30) observable colonies was quantified using viable plate count, the number of colony forming unit per 100 ul was estimated. The experimental colony counts at the beginning of the biodegradation and after 48 h of incubation were compared. Regarding sample 1, initially, *K. pneumoniae* was more abundant than *A. baumannii*. However, as the CIP and LEV biodegradation process proceeded, the ratio became the same as shown in Fig. 4a, b. On the contrary, the initial ratio between the bacterial isolates in sample 2 (*K. pneumoniae* and *E. miricola*) was practically the same, but as the biodegradation process proceeded, the ratio was changed until *E. miricola* became significantly predominate over *K. pneumoniae* as shown in Fig. 4c, d.

### The aac (6′)-Ib-cr variant of aminoglycoside acetyl-transferase involved in FQs degradation is present in the individual isolates of both consortia

**Fig. 5:**
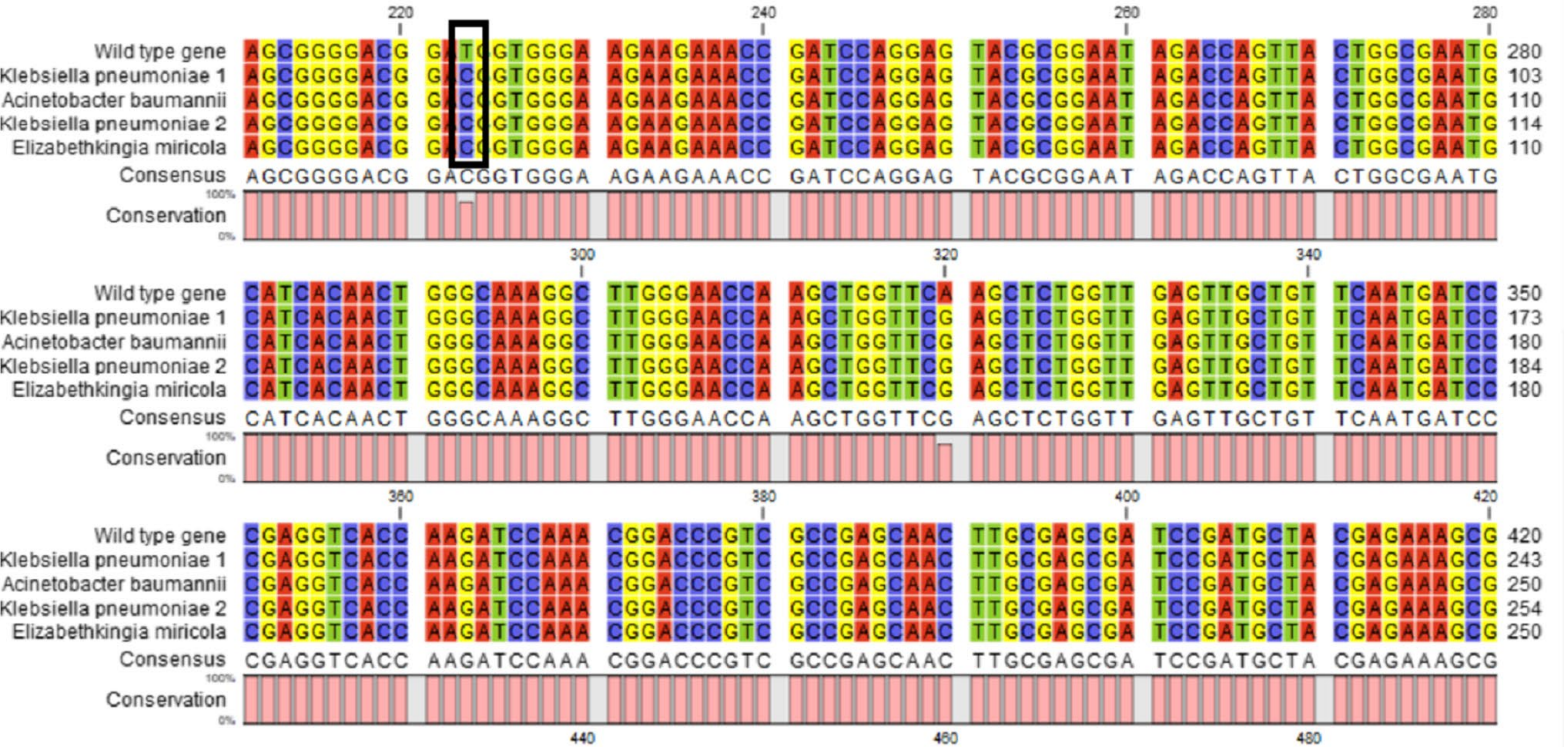
Alignment of the nucleotide sequence of the four isolates against the *aac(6′)-Ib* gene. The pink bars, representing the % conservation of the nucleotides, are showing high conservation % among different strains. The variation in the nucleotide sequence at the Bstf5l restriction site (GGATGGTGG) of the gene *aac(6′)-Ib-cr* (T → C) in the four isolates and the wild type is highlighted. The alignment was generated by the CLC Workbench 8.5 software

The individual bacterial isolates in both consortia were molecularly screened for *aac (6 ') –Ib-cr* gene that is involved in the degradation of FQs. All four isolates were positives for the gene. The *aac(6′)-Ib-cr* variant was distinguished from the wild type gene by direct sequencing and confirming the absence of the restriction site of BstF5I found in the wild-type gene (Fig. 5).

### 1-aminobenzotriazole abolishes completely the biodegradation activity of FQs

**Fig. 6:**
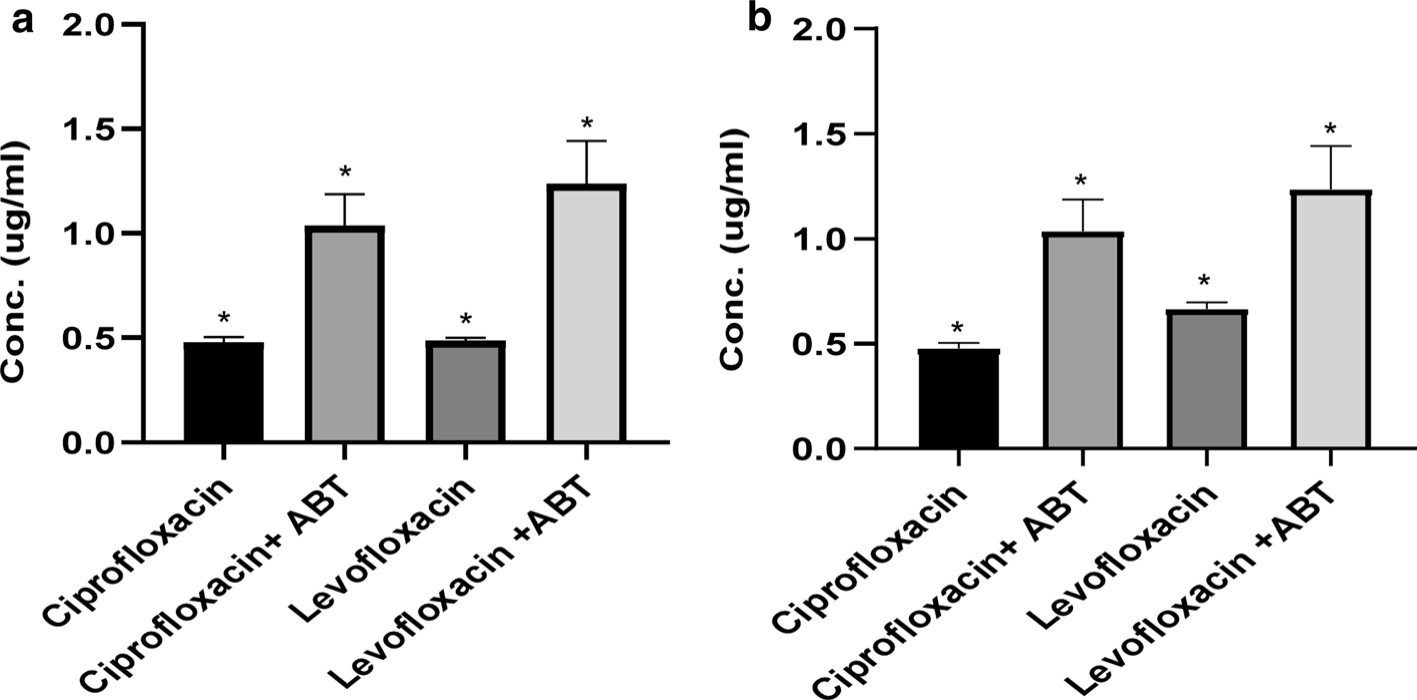
The 1-aminobenzo triazole diminishes the biodegradation of CIP and LEV. MMSM was supplemented with CIP or LEV as a sole carbon source. Experiments were conducted under the optimum degradation conditions (30 °C, pH = 5, 180 rpm). **a** A comparison between the biodegradation rate of CIP and LEV (1 mg L^−1^) by sample 1 (*K. pneumoniae* and *A. baumannii*) in the presence and absence of CYP 450 inhibitor (ABT). **b** A comparison between the biodegradation rate of CIP and LEV (1 mg L^−1^) by sample 2 (*K. pneumoniae* and *E. miricola*) in the presence and absence of CYP 450 inhibitor (ABT). Error bars represent SD (n = 3). The data were analyzed by ANOVA’s test using graph pad prism version 8 programme (USA). The *indicates significant differences, *p* < 0.05. The figure was generated using CANVA X GIS programme

The concentration of CIP and LEV was measured by HPLC–DAD after 48 h of incubation in absence and presence of 1-aminobenzotriazole. Complete inhibition of the biodegradation of CIP and LEV by sample 1 (Fig. 6a) and sample 2 (Fig. 6b) in the presence of 1-aminobenzotriazole indicated the possible involvement of CYP450 enzymes.

### CIP and LEV metabolites are non-phytotoxic

**Fig. 7:**
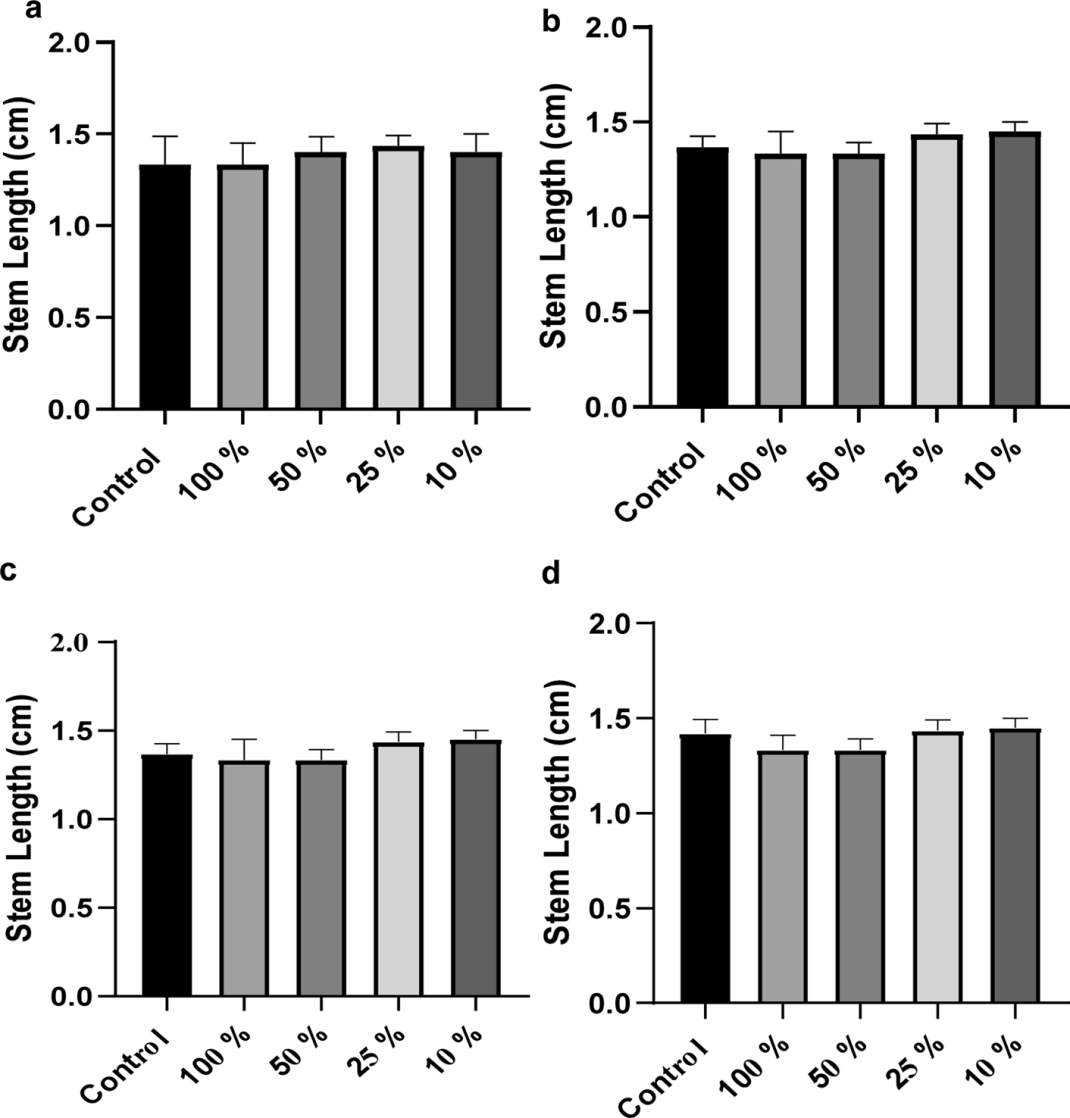
The CIP and LEV biodegradation metabolites exhibit no toxicity levels**.** The average stem lengths were measured in the presence of biodegradation metabolites. **a** A comparison between the recorded averages of stem length of *L. sativum* seeds of biodegradation metabolites of CIP by Sample 1 (*K. pneumoniae* and *A. baumannii*) and control. **b** A comparison between the recorded averages of stem length of *L. sativum* seeds of biodegradation metabolites of LEV by Sample 1 (*K. pneumoniae* and *A. baumannii*) and control. **c** A comparison between the recorded averages of stem length of *L. sativum* seeds of biodegradation metabolites of CIP by Sample 2 (*K. pneumoniae* and *E. miricola*) and control. **d** A comparison between the recorded averages of stem length of *L. sativum* seeds of biodegradation metabolites of LEV by Sample 2 (*K. pneumoniae* and *E. miricola*) and control. The data were analyzed by ANOVA’s test using graph pad prism version 8 programme (USA)

The phytotoxicity of CIP and LEV metabolites by Sample 1 (*K. pneumoniae* and *A. baumannii*) (Fig. 7a, b) and by sample 2 (*K. pneumoniae* and *E. miricola*) (Fig. 7c, d) was assessed using *L. sativum.* Each sample included 15 seeds of *L. sativum*, distributed on filter paper in Petri dishes. The length of the seeds stems was measured. Analysis of variance (ANOVA) was performed to compare the inhibitory effect of CIP and LEV metabolites solutions at the concentrations mentioned above with MMSM only as control group. No statistically significant differences were found between the tested and the control groups (the difference is considered significant if *p* value < 0.05).

## Discussion

In recent years antimicrobials have been viewed as emerging contaminants due to their continued incidence and persistence, as well as their widespread in natural and aquatic ecosystems, even in low concentrations (Frade et al. 2014). FQs have been observed in the environment around the world, indicating that FQs elimination using traditional methods from wastewater and water is ineffective and it’s believed to pose health risks to humans and other living organisms. In this study, two FQs antibiotics, CIP hydrochloride and LEV, were used as the only sole source of carbon in biodegradation tests.

Various microorganisms had been studied for their capabilities to degrade aromatic compounds (Amorim et al. 2014; Maia et al. 2018). In our study, two bacterial consortia were able to biodegrade both CIP and LEV; the first bacterial consortium was composed of *A. baumannii* and *K. pneumoniae.* Among the microbial communities involved in various ecosystems, i.e. soil, fresh/sewage water, and solid waste, various strains of the genus *Acinetobacter* have attracted a surge interest from a medical, environmental and biotechnological point of view. *A. baumannii* is abundant in nature, and can be acquired from water, soil and living organisms. Some strains of *Acinetobacter* are known to be associate in biodegradation of different number of pollutants such as Permethrin (Zhan et al. 2018), phenanthrene, 3-chloroaniline (Duc 2016), diesel (Ho et al. 2020), indole (Sadauskas et al. 2017), malathion (Xie et al. 2009), deltamethrin (Tang et al. 2020), azo dye (Sreedharan et al. 2021) and phenol (Liu et al. 2020). Biodegradation of cellulose, chitin, pectin, fluoranthene, and phenanthrene by *Actinetobacter* from deep Sea Sediments were reported by (Chen et al. 2016). Bioremediation of petroleum hydrocarbons by *Acinetobacter* were described in (Cai et al. 2021). In addition, some of the *Acinetobacter* strains are also recognized to produce important by-products. There are countless applications of *Acinetobacter* strains in the treatment of dangerous waste or as generators of economically beneficial bio-products.

*Klebsiella* species are among the well-studied microbes both in medicine field, as ones of the most resilient opportunistic pathogens, and in industry, due to their promising biochemical properties. *klebsiella* strains were especially investigated for degradation of polycyclic aromatic hydrocarbons kerosene (Ali et al. 2018), petroleum (Bilen Ozyurek and Bilkay 2017), diclofenac (Stylianou et al. 2018), atrazine (Zhang et al. 2019), organochlorine insecticide (Kwon et al. 2005), 3-phenoxybenzoic acid and pyrethroid pesticides (Tang et al. 2019) and nitro aromatic compounds (Boopathy and Melancon 2004). *K. pneumoniae* was reported in the removal of penicillin G from environment by (Wang et al. 2014). A microbial consortium containing *K. pneumoniae* was able to biodegrade two FQs norfloxacin and ofloxacin (Jałowiecki et al. 2019). *K. pneumoniae* is studied also for the elimination of heavy metals or metalloids from contaminated sites such as lead (Atikpo and Ihimekpen 2020).

The second consortium is composed of *E. miricola.* In 1959 Elizabeth O. King discovered the *Elizabethkingia* species. They are aerobic, non-fermenting, gram negative, and non-motile bacteria abundant in the environment (King 1959). The genus at the beginning was classified as *Flavobacterium*, then in 1994, it was specified as *Chryseobacterium* (Vandamme et al. 1994). It was assigned to the new genus *Elizabethkingia* in 2005 based primarily on the sequencing of 16S rRNA gene (Kim et al. 2005), it comprise a group of environmental bacteria that is widely found in water, soil and plants, including adequately chlorinated municipal water supplies systems (Kirby et al. 2004). *E. meningoseptica, E. anophelis, and E. miricola* have been recognized for their medical importance (Peng et al. 2020).There have been no reported studies on the use of *E. miricola* in bioremediation until now.

Regarding our study, in sample 1, the MIC of CIP and LEV for *K. Pneumoniae* and *A. Baumannii* was 16 mg L^−1^ and 8 mg L^−1^ respectively, while the MIC of CIP and LEV for *K. Pneumoniae* in sample 2 was 16 mg L^−1^ and 8 mg L^−1^ respectively, and for *E. miricola* was 8 mg L^−1^ for both FQs, which coincides with the maximum concentration of FQs that sample 1 and sample 2 can degrade as a sole carbon source (8 mg L^−1^).

The relative abundance between isolates of the two bacterial consortia during the biodegradation of CIP and LEV was investigated. The data revealed that in sample 1 initially, *K. pneumoniae* was more abundant than *A. baumannii*. However, as the CIP and LEV biodegradation process progressed, the ratio became the same. Interestingly, the initial ratio between the bacterial isolates in sample 2 (*K. pneumoniae* and *E. miricola*) was essentially the same, but as the biodegradation process proceeded, the ratio was changed and *E. miricola* became the foremost abundant one. Accordingly, in order to clarify this point, we need to highlight that only two single bacterial strains were documented for their ability to biodegrade CIP. *Labrys Portucalensis* reported by (Amorim et al. 2014) was able to degrade FQs in the presence of an easily degradable carbon source, while (Pan et al. 2018) reported the biodegradation of CIP by *Thermus thermophilushas* at 70 °C. However, in our study the effect of temperature had no significant effect on the biodegradation process. Probably due to the fact that the biodegradation/biotransformation of recalcitrant pollutants such as FQs in natural environments cannot be easily performed by a single microorganism (Rusch et al. 2019), we hypothesize that in each consortium one of the microorganisms (*K. pneumonia*) initiates the biodegradation of CIP or LEV producing metabolites that can be then used by the other bacterium (*A. baumannii* and *E. miricola)* which is confirmed by the inability of the pure culture of bacterial isolates to biodegrade FQs. However, further investigations are needed to acquire further knowledge and evidence.

There had been several studies reporting the biodegradation of CIP as the only source of carbon (Amorim et al. 2014; Liao et al. 2016; Pan et al. 2018). On the contrary, only few documented studies reporting the biodegradation of LEV as the only carbon source are available (Maia et al. 2018; Shu et al. 2021). For further knowledge about the biodegradation of CIP and LEV, the biodegradation metabolites of the two compounds throughout the experiment were investigated. Seven degradation metabolites were revealed by LC-MS-MS in case of CIP. The first compound, denoted as CIP-A (N-acetyl ciprofloxacin), was formed by acetylation of NH group in piperazine ring. This reaction is known to be catalyzed by the N acetyl transeferase enzyme, which coincides with the data showed by both (Jung et al. 2009; Robicsek et al. 2006) who reported the role of such enzyme in CIP biodegradation. Another compound, CIP-B (Ethylene-N-ciprofloxacin) was formed as a result of oxidation of the piperazinyl substituent. A net loss of C_2_H_2_ at the piperazinyl substituent of CIP lead to the formation of CIP-B that appeared in sample 2 (*E. miricola* and *K. pneumoniae)*. The same compound was previously reported as a CIP bacterial degradation metabolite by *Labrys portucalensis*, Sulfate reducing bacteria and *Thermus thermophilus* bacterium, respectively (Amorim et al. 2014; Jia et al. 2018; Maia et al. 2014; Pan et al. 2018) and also by the brown-rot fungus *Gloeophyllu striatum* (Wetzstein et al. 1999). CIP-B was also recognized as a mutual metabolite in mammals with insignificant antibacterial effect (Dalhoff and Bergan 1998; Wetzstein et al. 1999). CIP-C (N-formyl ciprofloxacin), detected in both consortia, was also revealed by (Pan et al. 2018) as one of CIP biodegradation products. CIP-D (mono-hydroxylated‐de-fluorinated CIP) was formed by the substitution of the fluorine atom with hydroxyl group. CIP-D was previously reported by (Jia et al. 2018; Liao et al. 2016) as one of the biodegradation products of CIP by Sulfate reducing bacteria, and *Gammaproteobacteria, Bacteroidia, Betaproteobacteria* bacterium, respectively. CIP-E was first reported by (Pan et al. 2018) in biodegradation of CIP by *Thermus thermophilus* as 7-amino-6-fluoro-4-oxo-1,4-dihydroquinoline-3-carboxylic acid. Metabolites CIP-F and CIP-G as di and mono hydroxylated ciprofloxacin were proposed by (Wetzstein et al. 1999) in which both compounds were formed by undergoing hydroxylation, these compounds were only reported due to fungal degradation of CIP. Interestingly, this study is the first to report the mono and di hydroxylated ciprofloxacin as bacterial biodegradation products. To that end, we can assume that hydroxylation is a crucial step for FQs biodegradation, which may occur at varies locations on the quinolone ring throughout CIP and LEV biodegradation by bacteria (Amorim et al. 2014) and fungi (Prieto et al. 2011; Wetzstein et al. 1999).

As for LEV, Potential degradation metabolites were also proposed in this study. Seven compounds were generated during LEV degradation. LEV-A was formed by decarboxylation and formation of N-oxide to LEV. LEV-B was formed by N-demethylation of the N-methylpiperazine moiety, this compound was previously revealed by (Shu et al. 2021) as one of LEV degradation metabolites produced by *lactobacillus*. LEV-C was proposed to be formed by decarboxylation reaction, which is also reported by (Xiong et al. 2017) during degradation of LEV by freshwater green algae. LEV-D was obtained by morpholine ring breakage. Deflourination of LEV yielded LEV-E metabolite. LEV-F degradation product formed by defloration and demethylation followed by a hydroxylation reaction. LEV-G was formed by decarboxylation and N-demethylation that occur simultaneously leading to the removal of carbon dioxide and a methyl group.

AAC(6’)-Ib-cr (Park et al. 2006) and CrpP (Chávez-Jacobo et al. 2018) are the only two enzymes that has been described to possess CIP-modifying activity. Aminoglycoside N-acetyl-transferases (*aac(6')-Ib)*, the most ubiquitous aminoglycoside modifying enzyme, resistant to tobramycin and kanamycin (Vakulenko and Mobashery 2003), was first detected in *K. pneumoniae* isolates in 1986 (Tolmasky et al. 1986). There are two main subclasses of aac (6′) enzymes, which are discriminated based on the aminoglycosides inhibited aac*(6′)-I* and *aac(6′)-II* (Tolmasky et al. 1986; Woloj et al. 1986; Rather et al. 1992; Shaw et al. 1993; Tolmasky et al. 1986; Woloj et al. 1986) A variant of the aac*(6')-Ib* recognized as *aac(6')-Ib-cr* was identified by (Robicsek et al. 2006). This enzyme comprises FQs in addition to aminoglycosides as its drug targets to. In a study performed by Jung and coworkers (Jung et al. 2009), a strain of *E. coli*, obtained from a municipal waste water treatment plant was found to possess a variant of a gene that encodes an aminoglycoside acetyl transferase enzyme which can acetyl the piperazine ring, thereby inactivating certain FQs. However, other antimicrobials such as LEV are not altered by this enzyme due to the absence of the piperazinyl constituent in its structure in addition to the presence of methyl group. Although since 1986 about 30 variants of this gene have been revealed, the two base-pair alteration accountable for the CIP transformation are distinctive to this variant (Wetzstein et al. 1999). Regarding our study, since the analysis of degradation metabolites by LC-MS-MS showed the presence of N-acetyl ciprofloxacin compound, the bacterial isolates of each consortium were screened for the presence of *aac(6′)-Ib-cr* and the presence of *aac(6′)-Ib-cr* gene was confirmed in all isolates.

The CrpP enzyme reported by (Chávez-Jacobo et al. 2018) showed enzymatic activity against CIP through phosphorylation producing a CIP ATP compound. In our study the in vitro CIP metabolites analysis using LC-MS-MS did not show any evidence on the presence of a molecular ion that correlates to CIP-ATP or any of its fragmented compounds. Thus, we ruled out the role of the CrpP enzyme in CIP biodegradation in our bacterial consortia.

In order to study the role of CYP 450 in the biodegradation of FQs, 1-aminobenzotriazole (ABT) was introduced to the media as it’s considered a pan in-activator of the xenobiotic metabolizing forms of cytochrome P450 in animals, plants, insects, and microorganisms (de Montellano 2018). The strong inhibition of the process indicated the crucial role played by CYP450 in FQs biodegradation in concise with data presented by Jia and coworkers (Jia et al. 2018), who reported that throughout CIP biodegradation in a sludge system of sulfate-reducing anaerobic bacteria, the addition of ABT as a CYP450 inhibitor had significantly inhibited the biodegradation process. Therefore, the CYP450 having an essential part in FQs biodegradation was suggested by the authors.

The results of the phytotoxicity assay revealed that the two bacterial consortia can be used in the bioremediation of CIP and LEV yielding non phytotoxic compounds, thus, providing an environmentally safe approach for dealing with FQs environmental pollution.

## Conclusion

This Study is the first to report the biodegradation activity of *E. miricola*, *K. pneumoniae and A. baumannii* towards FQs. For CIP biodegradation, the main steps are demethylation, acetylation, formylation, and hydroxylation. Only few studies had reported about the enzymes and pathways involved in the biodegradation of CIP considering the numerous number of biodegradation metabolites of CIP. On the other hand, the principle steps of LEV biodegradation are deflourination, hydroxylation, demethylation, and decarboxylation.Our findings indicate the crucial role played by FQs aminoglycoside acetyl-transferase and CYP450 enzymes in the biodegradation of FQs. Taking this all together suggests that the results of this research can help to develop suitable bio-augmentation strategies for better FQs wastewater treatment processes.

## Supplementary Information

Below is the link to the electronic supplementary material.

Supplementary file1 (DOCX 478 kb)

## Data Availability

The data that support the findings of this study are available from the corresponding author upon reasonable request.
