# Supplementary material for: Acinetobacter baumannii, Klebsiella pneumoniae and Elizabethkingia miricola isolated from wastewater have biodegradable activity against fluoroquinolone
Source: World J Microbiol Biotechnol. 2022 Aug 16;38(11):187. doi: 10.1007/s11274-022-03367-5 (PMC9381475; doi:10.1007/s11274-022-03367-5)
Supplement: Supplementary file 1 — Supplementary file1 (DOCX 478 kb) [file 11274_2022_3367_MOESM1_ESM.docx]

**Supplementary material**

Article title: *Acinetobacter baumannii*, *Klebsiella pneumoniae* and *Elizabethkingia miricola* isolated from wastewater have biodegradable activity against fluoroquinolone

Journal name World Journal of Microbiology and Biotechnology

Authors: Reham Alaa Eldin Shaker^1^, Yosra Ibrahim Nagy^1^, Mina E. Adly^2^, Rania Abdelmonem Khattab^1^, Yasser M. Ragab^1^

^1^ Department of Microbiology and Immunology, Faculty of Pharmacy, Cairo University, Kasr Al-Aini 11562, Cairo, Egypt

^2^ Department of Pharmaceutical Organic Chemistry, Faculty of Pharmacy, Cairo University, Kasr Al-Aini 11562, Cairo, Egypt


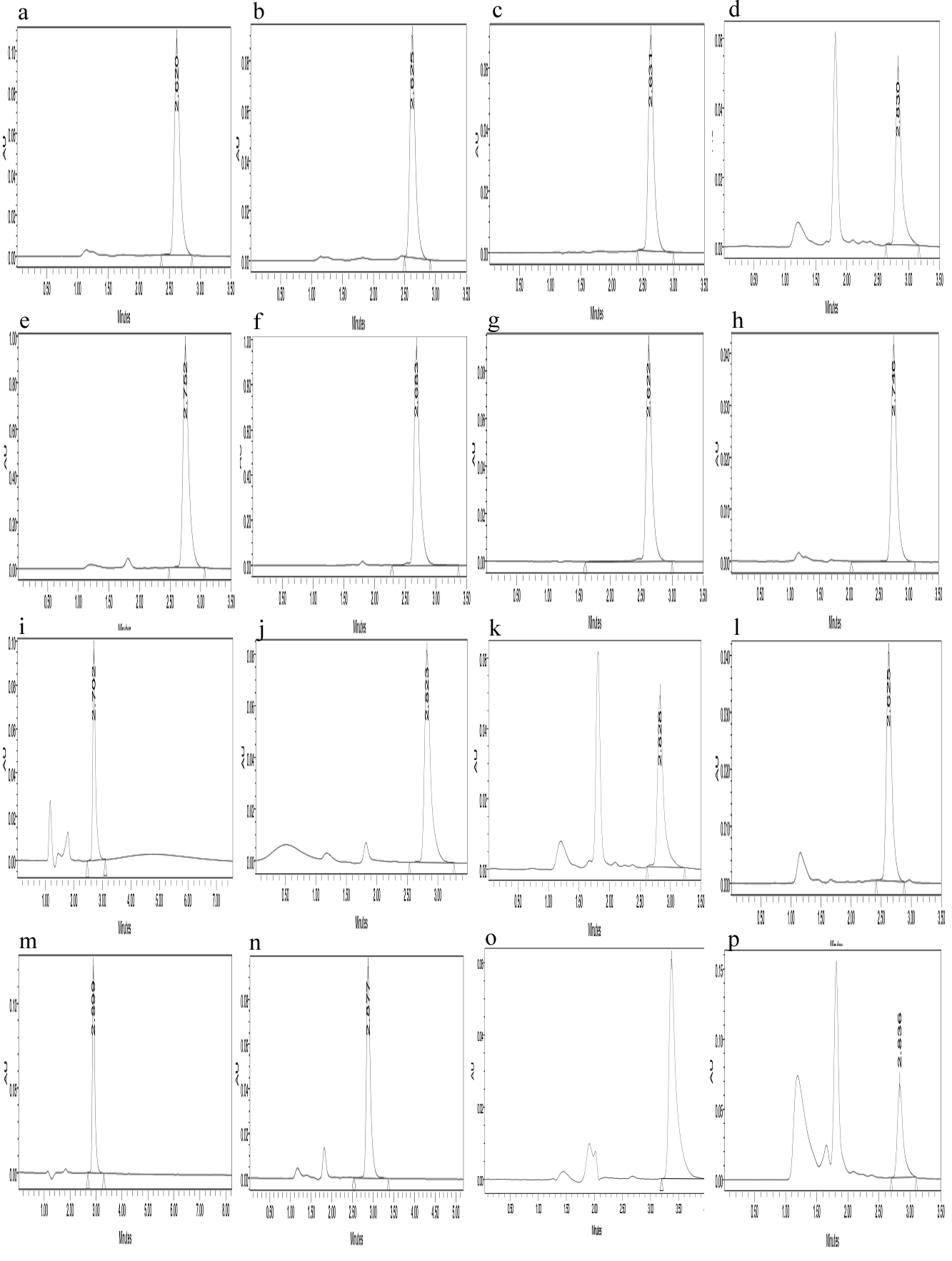
E-mail address: [yosra.nagy@pharma.cu.edu.eg](mailto:yosra.nagy@pharma.cu.edu.eg)


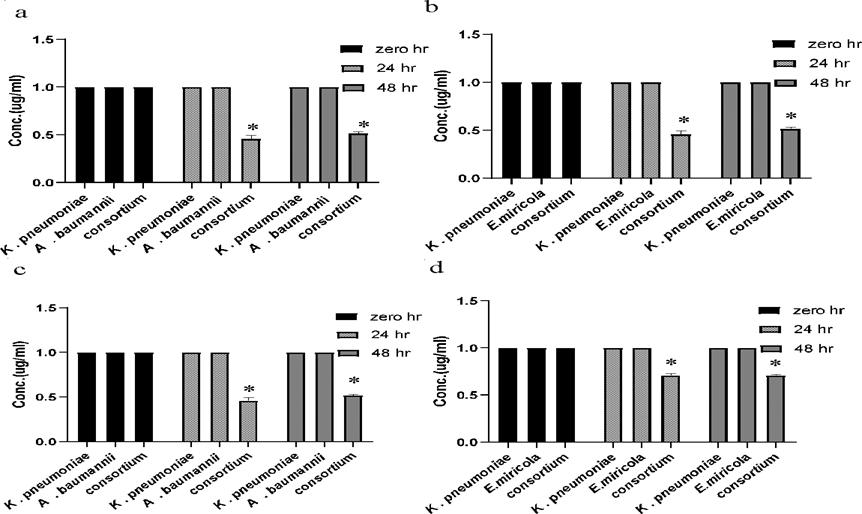
**Fig. S1** HPLC chromatogram of the CIP and LEV biodegradation**.** The concentrations of CIP and LEV were calculated by relative peak area. Calibration curves for CIP and LEV were constructed by plotting the relative peak area of FQs versus concentration. [2a-2d] The chromatograms of the HPLC analyses for the CIP (1 mg L^-1^) by sample 1 **(***K. pneumoniae* and *A. baumannii*) at zero, 12, 24 and 48 hr, respectively (CIP tr – 2.6-2.8 min). [2e-2h] The chromatograms of the HPLC analyses for the CIP (1 mg L^-1^) by sample 2 (*K. pneumoniae* and *E. miricola*) at zero, 12, 24 and 48 hr, respectively (CIP tr – 2.6-2.8 min). [2i-2l] The chromatograms of the HPLC analyses for the LEV (1 mg L^-1^) by sample 1 (*K. pneumoniae* and *A. baumannii*) at zero, 12, 24 and 48 hr, respectively (LEV tr – 2.7-2.8 min). [2m-2p] The chromatograms of the HPLC analyses for the LEV (1 mg L^-1^) by sample 2 (*K. pneumoniae* and *E. miricola*) at zero, 12, 24 and 48 hr, respectively (LEV tr – 2.7-2.8 min)


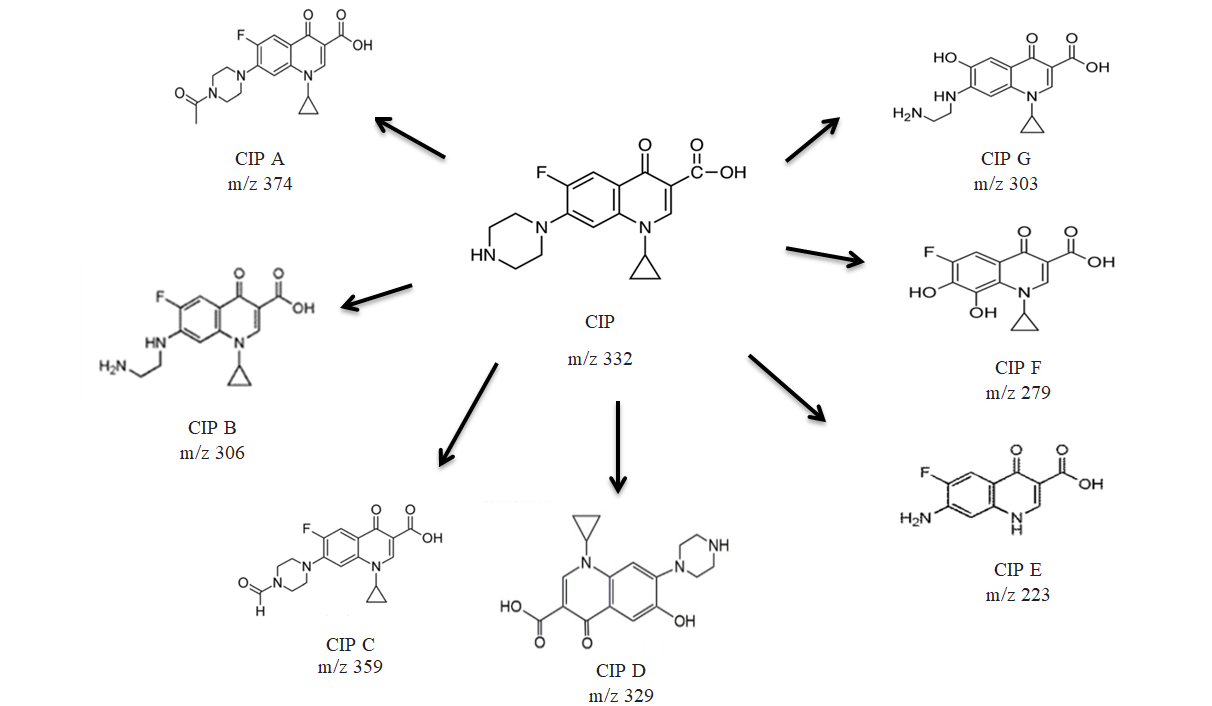
**Fig. S2** The pure cultures of the individual bacterial isolates are unable to biodegrade CIP and LEV. MMSM was supplemented with CIP or LEV as a sole carbon source. Experiments were conducted under the optimum degradation conditions (30 °C, pH= 5, 180 rpm). [a] and [b] A comparison between the biodegradation rate of CIP and LEV (1mg L^-1^) by the individual bacterial isolates of sample 1 and the consortium (*K. pneumoniae* and *A. baumannii*). [c] and [d] A comparison between the biodegradation rate of CIP and LEV (1mg L^-1^) by the individual bacterial isolates of sample 2 and the consortium (*K. pneumoniae* and *E. miricola*). Values are the means of three independent experiments. Error bars represent standard deviations (n= 3). The analysis was conducted using two way ANOVA’s test using graph pad prism version 8 programme (USA). The * indicates *p* < 0.05.

**Fig. S3** Structures and mass spectral data for ciprofloxacin and postulated biodegradation metabolites as determined from liquid chromatography tandem mass for the two samples. Biodegradation products generated by sample 1 were [CIP C, E, F, and G] and sample 2 [CIP
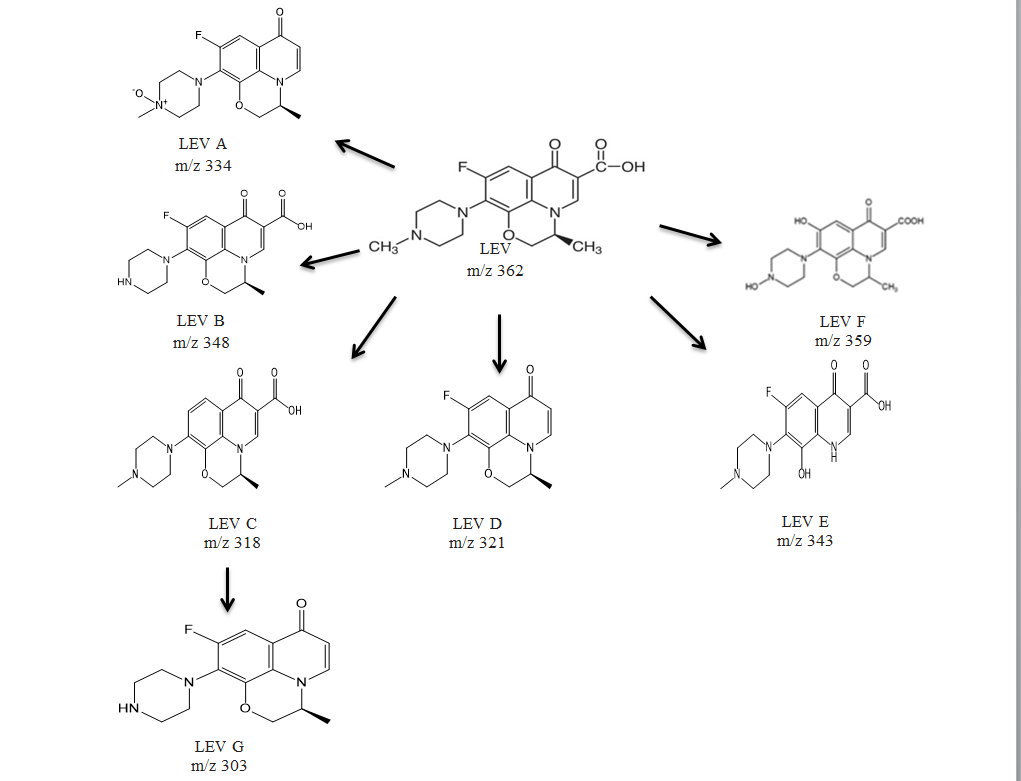
A, B, C, and D].

**Fig. S4** Structures and mass spectral data for levofloxacin and postulated biodegradation metabolites as determined from liquid chromatography tandem mass for the two samples. Biodegradation products generated by sample 1 were [LEV A, B, C, D, E, F and G] and sample 2 [LEV A, B, C, D, E and F].
